# Supplementary material for: Content-rich biological network constructed by mining PubMed abstracts
Source: BMC Bioinformatics. 2004 Oct 8;5:147. doi: 10.1186/1471-2105-5-147 (PMC528731; doi:10.1186/1471-2105-5-147)
Supplement: Additional File 2 — The original results of the above study (non-essential files are deleted to keep the file size under the limit set by BMC bioinformatics). [file 1471-2105-5-147-S2.bz2 › chilibotAdditionalFile2/dip40/438ID9430724E10383/html/CCR4_CCL22.html]

 


 **CCR4** and **CCL22** 
  
Found 85 abstracts in PubMed, retrieved 40.  
 

 What does Google say? 
 PDF only 
| .edu only 

---

**Interactive relationship** (e.g. stimulation, inhibition, etc)

**Neutral relationship**- Multiple determinants are involved in HIV coreceptor use as demonstrated by  **CCR4**   **CCL22**  interaction in peripheral blood mononuclear cells PBMCs.  Ref: 12429730 J Leukoc Biol, 2002
- Since the ligand of the TARC and MDC  [ **CCL22** ]  is  **CCR4** , which is specifically expressed on Th2 type T cells, the production of these CC chemokines is likely to play important roles in the T cell and B cell interaction.  Ref: 12715916 J Med Dent Sci, 2003
- The helper Th 2 cell attracting chemokines thymus and activation regulated chemokine TARC and macrophage derived chemokine  [ **CCL22** ]  MDC  [ **CCL22** ]  are ligands for the chemokine receptor  **CCR4** .  Ref: 12645858 J Clin Immunol, 2003
- ... interactions of  **CCR4**  with MDC  [ **CCL22** ]   **CCL22**  or TARC CCL17, CCR8 with I 309 CCL1, and CRTH2 with prostaglandin D 2 play a critical role in the allergen induced recruitment of Th2 cells in the target tissues of allergic inflammation.  Ref: 12009564 Mol Immunol, 2002
- Type 2 T cells have been shown to be preferentially recruited by the chemokines eotaxin CCL11, macrophage derived chemokine  [ **CCL22** ]  MDC  [ **CCL22** ] ,  **CCL22** , and thymus activation regulated chemokine CCL17 through their interaction with CCR3 and  **CCR4** , respectively.  Ref: 12023397 J Immunol, 2002
- However, T cells which are CD4 CD152  **CCR4**  migrate selectively in vitro in response to the chemokine  **CCL22**  specific for the receptor  **CCR4**  produced by the malignant B cells and are always seen amongst the malignant cells in bone marrow and lymph nodes from B CLL patients.  Ref: 12688308 Leuk Lymphoma, 2003
- Moreover,  **CCR4**  and CXCR6 were preferentially induced in T cells activated by mDC  [ **CCL22** ]  and pDC, respectively.  Ref: 12645946 Eur J Immunol, 2003
- Surprisingly, only the  **CCL22**  analogue with the biotin in position K66 constituted a suitable staining reagent for  **CCR4**  positive cells.  Ref: 12857385 J Biomol Screen, 2003
- MDC  [ **CCL22** ]  and were closely surrounded by memory type T cells expressing its receptor,  **CCR4** .  Ref: 12474232 J Pathol, 2003
- The dominance of  **CCL22**  over CCL17 extends to other  **CCR4**  mediated phenomena such as receptor desensitization and internalization and correlates with the peculiar kinetics of  **CCR4**  engagement by the two ligands.  Ref: 12193695 J Immunol, 2002
- Thymus and activation regulated chemokine TARC CCL17 and macrophage derived chemokine  [ **CCL22** ]  MDC  [ **CCL22** ]   **CCL22**  are a pair of CC chemokines known to selectively attract T h 2 type memory T cells via  **CCR4** .  Ref: 12096036 Int Immunol, 2002
- T cells expressing CLA and  **CCR4**  were also found at high levels in cutaneous T cell lymphoma lesions along with abundant expression of the two  **CCR4**  ligands TARC CCL17 and MDC  [ **CCL22** ]   **CCL22** .  Ref: 12485447 J Invest Dermatol, 2002
- Recently, macrophage derived chemokine  [ **CCL22** ]  MDC  [ **CCL22** ]   **CCL22** , a CC chemokine, was identified as a selective chemoattractant for CC chemokine receptor 4  [ **CCR4** ]   **CCR4**  expressing cells, in addition to thymus and activation regulated chemokine TARC.  Ref: 11876749 Clin Exp Immunol, 2002
- CC chemokine receptor 4  [ **CCR4** ]   **CCR4**  has been identified as a specific receptor for both thymus and activation regulated chemokine TARC and macrophage derived chemokine  [ **CCL22** ]  MDC  [ **CCL22** ] , and is preferentially expressed on Th2 cells.  Ref: 12757454 Allergy, 2003
- Three chemokines in particular, CXCL12 SDF 1, CCL17 TARC, and  **CCL22**  MDC  [ **CCL22** ] , recently have been shown to be strong and rapid activators of platelet aggregation and adhesion after their binding to platelet CXCR4 or  **CCR4** , when acting in combination with low levels of primary agonists.  Ref: 12851650 Microcirculation, 2003
- Interestingly, neutralization of macrophage derived chemokine  [ **CCL22** ]  MDC  [ **CCL22** ]  with Ab caused a significant reduction of  **CCR4**  positive T cells within the pancreatic infiltrates and inhibited the development of insulitis and diabetes.  Ref: 12464673 J Clin Invest, 2002
- Fractalkine, SDF 1alpha, RANTES and MDC  [ **CCL22** ]  inhibited the I Ba in CX3CR1, CXCR4, CCR5 and  **CCR4**  expressing G1A1 cells, respectively.  Ref: 11880151 J Neuroimmunol, 2002
- Furthermore, enhanced recruitment of  **CCR4**  bearing cells in NOD mice resulting from transgenic expression of MDC  [ **CCL22** ]  resulted in acceleration of clinical disease.  Ref: 12464673 J Clin Invest, 2002

**Non-interactive relationship** (e.g. studied together, co-existance, homology, etc.)

- The aims of this study were to examine the expression of three recently described potent T lymphocyte chemoattractants, PARC pulmonary and activation regulated chemokine, macrophage derived chemokine  [ **CCL22** ]  MDC  [ **CCL22** ] , and I 309, the MDC  [ **CCL22** ]  receptor  **CCR4** , and T lymphocyte activation markers, CD25, CD26, CD62L, CD71, and CD30, and to correlate them with the counts of CD3 T lymphocytes in the conjunctiva of patients with VKC.  Ref: 12234902 Br J Ophthalmol, 2002
- In view of the potential importance of  **CCR4**  in skin homing of T cells, we investigated the expression pattern of  **CCR4**  and its ligands TARC CCL17 and MDC  [ **CCL22** ]   **CCL22**  in the peripheral blood and skin of patients with cutaneous T cell lymphoma, a putative malignancy of the skin homing T cells.  Ref: 12485447 J Invest Dermatol, 2002
- Among  **CCR4**  ligands, skin vessels endothelial cells present C C chemokine ligand CCL 17 but not  **CCL22**  consistent with CCL17 involvement in T lymphocyte arrest on endothelial cells.  Ref: 12193695 J Immunol, 2002
- which express high levels of  **CCR4**  with  **CCL22**  caused down modulation of endogenous  **CCR4**  but had no significant effect on HIV 1 entry, suggesting that  **CCR4**  may not be used as an entry coreceptor.  Ref: 12429730 J Leukoc Biol, 2002
- We analyzed the functional role of macrophage derived chemokine  [ **CCL22** ]  MDC  [ **CCL22** ] , a potent mononuclear cell chemoattractant, during the progression of anti glomerular basement membrane GBM antibody Ab GN, a model of crescentic GN in the WKY rat, and whether the effects of MDC  [ **CCL22** ]  were dependent on its receptor  **CCR4** .  Ref: 12651599 Am J Pathol, 2003
- CONCLUSIONS Our data suggest that Th2 cells, which express  **CCR4**  and its ligands TARC and MDC  [ **CCL22** ] , contribute to the pathogenesis of EP in the lung.  Ref: 12757454 Allergy, 2003
- By contrast, expression of  **CCR4**  and responsiveness to  **CCL22**  is rapidly induced at the early stages of both Th1 Th2 cell development.  Ref: 11981813 Eur J Immunol, 2002
- In this study, we investigate the effects of VIP PACAP on two chemokines, i.e. CXCL10 IP 10 acting on CXCR3 expressed on activated Th1 cells, and  **CCL22**  MDC  [ **CCL22** ]  acting on  **CCR4**  and 8 expressed on activated Th2 cells.  Ref: 12446011 J Neuroimmunol, 2002
- This was associated with the expression of their agonistic receptor CXCR3, while mRNA for TARC and MDC  [ **CCL22** ] , and their receptor  **CCR4**  were not detected.  Ref: 11912011 J Dermatol Sci, 2002
- Macrophage derived chemokine  [ **CCL22** ]  MDC  [ **CCL22** ]   **CCL22**  and its receptor  **CCR4**  have been implicated in chronic inflammatory processes and in the homing of monocytes, Th2 cells and regulatory T cell subsets.  Ref: 12225884 J Neuroimmunol, 2002
- METHOD Conjunctival biopsy specimens from 11 patients with active VKC, and eight control subjects were studied by immunohistochemical techniques using a panel of monoclonal and polyclonal antibodies directed against PARC, MDC  [ **CCL22** ] , I 309,  **CCR4** , CD25, CD26, CD62L, CD71, and CD30.  Ref: 12234902 Br J Ophthalmol, 2002
- There was a significant correlation between the number of  **CCR4**  CD4 T cells and the levels of TARC, MDC  [ **CCL22** ] , and IL 5 in BALF of patients with EP.  Ref: 12757454 Allergy, 2003
- In this study, we also found that MDC  [ **CCL22** ]  activity on Mo Mphi in this GN was at least partly dependent on a new variant of  **CCR4** .  Ref: 12651599 Am J Pathol, 2003
- The coreceptor activity was sensitive to  **CCR4**  specific antibodies and to the  **CCR4**  specific chemokine ligand macrophage derived chemokine  [ **CCL22** ]  MDC  [ **CCL22** ]  chemokine ligand 22  [ **CCL22** ]   **CCL22** .  Ref: 12429730 J Leukoc Biol, 2002
- BACKGROUND Macrophage derived chemokine  [ **CCL22** ]  MDC  [ **CCL22** ]  is a Th2 type chemokine and its receptor CC chemokine receptor 4  [ **CCR4** ]   **CCR4**  is preferentially expressed on Th2 cells.  Ref: 12670721 J Dermatol Sci, 2003
- These supernatants induced the migration of activated CD4, CD40L T cells expressing the  **CCL22**  receptor,  **CCR4** .  Ref: 11981828 Eur J Immunol, 2002
- Type 1 chemokines CXC chemokine ligand CXCL 9 and CXCL10 monokine induced by IFN gamma and IFN gamma inducible protein of 10 kDa CXCR3 ligands, and type 2 chemokines CC chemokine ligand CCL 11 eotaxin CCR3 ligand, CCL17 thymus and activation regulated chemokine  **CCR4**  ligand and  **CCL22**  monocyte derived chemokine  **CCR4**  ligand were readily detectable in the plasma of most participants.  Ref: 12407016 Int Immunol, 2002
- Here, we demonstrate that MDC  [ **CCL22** ]  and  **CCR4**  mRNAs are expressed in the central nervous system CNS of mice developing relapsing remitting and chronic relapsing forms of experimental autoimmune encephalomyelitis EAE.  Ref: 12225884 J Neuroimmunol, 2002
- Furthermore, we detected strong signals for  **CCR4** , TARC, and MDC  [ **CCL22** ]  in ATL skin lesions by RT PCR.  Ref: 11861261 Blood, 2002
- In contrast to naive T cells, primed T cell arrest was largely inhibited by pertussis toxin, neutralization of the CC chemokine, macrophage derived chemokine  [ **CCL22** ]   **CCL22** , or by desensitization of the  **CCL22**  receptor,  **CCR4** .  Ref: 11673480 J Immunol, 2001
- Accumulation of  **CCR4**  expressing CD4 T cells and high concentration of its ligands TARC and MDC  [ **CCL22** ]  in bronchoalveolar lavage fluid of patients with eosinophilic pneumonia.  Ref: 12757454 Allergy, 2003
- Cells transfected with gpCCR4 migrated toward the human and murine orthologues of the  **CCR4**  ligands, macrophage derived chemokine  [ **CCL22** ]  and thymus and activation regulated chemokine.  Ref: 11724798 J Biol Chem, 2002
- and 4 an efficient migration of fresh ATL cells to the  **CCR4**  ligands, TARC CCL17 and MDC  [ **CCL22** ]   **CCL22** , in chemotaxis assays.  Ref: 11861261 Blood, 2002
- These facts suggest possible involvement of two  **CCR4**  specific ligandsi.e., thymus and activation regulated chemokine TARC and macrophage derived chemokine  [ **CCL22** ]  MDC  [ **CCL22** ] , in the pathogenesis of bronchial asthma via recruitment of TH2 cells to inflammatory sites.  Ref: 11929424 Allergy, 2002
